# Prevalence and risk factors of sarcopenia in idiopathic pulmonary fibrosis: a systematic review and meta-analysis

**DOI:** 10.3389/fmed.2023.1187760

**Published:** 2023-06-08

**Authors:** Jiaye Li, Ye Lu, Mingming Deng, Run Tong, Qin Zhang, Yiding Bian, Jinrui Miao, Zilin Wang, Xiaoming Zhou, Gang Hou

**Affiliations:** ^1^Department of Pulmonary and Critical Care Medicine, Center of Respiratory Medicine, National Center for Respiratory Medicine, National Clinical Research Center for Respiratory Diseases, China-Japan Friendship Hospital, Beijing, China; ^2^Department of Respiratory and Critical Care Medicine, Shengjing Hospital of China Medical University, Shenyang, Liaoning, China; ^3^Center for Diagnosis and Management of Pulmonary Vascular Diseases, Department of Cardiology, Fuwai Hospital, Chinese Academy of Medical Sciences (CAMS) and Perceiving, Beijing, China

**Keywords:** IPF, sarcopenia, risk factors, review, meta-analysis

## Abstract

**Background:**

Sarcopenia often occurs as a comorbidity in many diseases which ultimately affects patient prognosis. However, it has received little attention in patients with idiopathic pulmonary fibrosis (IPF). This systematic review and meta-analysis aimed at determining the prevalence and risk factors of sarcopenia in patients with IPF.

**Methods:**

Embase, MEDLINE, Web of Science, and Cochrane databases were searched using relevant MeSH terms until December 31, 2022. The Newcastle-Ottawa Scale (NOS) was used for quality assessment and data analysis were performed using Stata MP 17.0 (Texas, USA). A random effects model was adopted to account for differences between articles, and the *I*^2^ statistic was used to describe statistical heterogeneities. Overall pooled estimates obtained from a random effects model were estimated using the metan command. Forest plots were generated to graphically represent the data of the meta-analysis. Meta-regression analysis was used for count or continuous variables. Egger test was used to evaluate publication bias and, if publication bias was observed, the trim and fill method was used.

**Main results:**

The search results showed 154 studies, and five studies (three cross-section and two cohort studies) with 477 participants were finally included. No significant heterogeneity was observed among studies included in the meta-analysis (*I*^2^ = 16.00%) and our study's publication bias is low (Egger test, *p* = 0.266). The prevalence of sarcopenia in patients with IPF was 26% (95% CI, 0.22–0.31). The risk factors for sarcopenia in patients with IPF were age (*p* = 0.0131), BMI (*p* = 0.001), FVC% (*p* < 0.001), FEV1% (*p* = 0.006), DLco% (*p* ≤ 0.001), and GAP score (*p* = 0.003).

**Conclusions:**

The pooled prevalence of sarcopenia in patients with IPF was 26%. The risk factors for sarcopenia in IPF patients were age, BMI, FVC%, FEV1%, DLco%, and GAP score. It is important to identify these risk factors as early as possible to improve the life quality of patients with IPF.

## 1. Introduction

Sarcopenia is a disease characterized by progressive and systematic loss of skeletal muscle mass and a decline in muscle quality and function (1). It is recognized as a complication in chronic diseases such as cancer, chronic obstructive pulmonary disease and idiopathic pulmonary fibrosis (IPF) (2, 3). These diseases combined with sarcopenia carries a risk of adverse outcomes, such as physical disability, poor quality of life and death (4). IPF is a chronic, progressive respiratory disease which has been reported to co-occur with sarcopenia (5–9). Previous studies have not given enough attention to sarcopenia in patients with IPF, partly due to the median survival time of patients with IPF only being 2–4 years (10) but with the development of antifibrotic therapy, some patients with IPF may live longer (11, 12). Considering the adverse outcomes that sarcopenia can cause in patients with IPF on life time and life quality, it is necessary to increase the awareness of sarcopenia as a comorbidity.

The aim of this review and meta-analysis was to determine the prevalence of sarcopenia in patients with IPF, and to analyze and explore the risk factors of sarcopenia in patients with IPF.

## 2. Methods

### 2.1. Search strategy

This systematic review and meta-analysis have been conducted in accordance with the Preferred Reporting Items for Systematic Reviews and Meta-Analyses (PRISMA) Guidelines 2020 (13).

An electronic database search was conducted until December 31, 2022 in four electronic databases (PubMed, Embase, Cochrane Library, and Web of Science), mainly using the following subject heading and free-text terms: “Idiopathic Pulmonary Fibroses,” “Pulmonary Fibroses, Idiopathic,” “Idiopathic Fibrosing Alveolitis, Chronic Form,” “Fibrosing Alveolitis, Cryptogenic,” “Fibrocystic Pulmonary Dysplasia,” “Dysplasia, Fibrocystic Pulmonary,” “Fibrocystic Pulmonary Dysplasias,” “Pulmonary Dysplasia, Fibrocystic,” “Cryptogenic Fibrosing Alveolitis,” “Cryptogenic Fibrosing Alveolitides,” “Fibrosing Alveolitides, Cryptogenic,” “Pulmonary Fibrosis, Idiopathic,” “Familial Idiopathic Pulmonary Fibrosis,” “Idiopathic Pulmonary Fibrosis, Familial,” “Usual Interstitial Pneumonia,” “Usual Interstitial Pneumonia,” “Usual Interstitial Pneumonias,” “Interstitial Pneumonitis, Usual,” “Pneumonitides, Usual Interstitial,” “Pneumonitis, Usual Interstitial,” “Usual Interstitial Pneumonitides,” “Usual Interstitial Pneumonitis,” “Sarcopenia,” “Sarcopenias,” “Muscle atrophy,” “Sarcopenic,” “Muscle attenuation,” “Muscle loss,” “Muscle depletion”. Only studies in English were included, and searches were limited to human studies. The detailed search strategies are shown in Supplementary material.

### 2.2. Selection criteria

The inclusion criteria were as follows: (1) studies reporting the association between sarcopenia and IPF; (2) studies using valid and objective methods to diagnose IPF rather than self-reporting; (3) observational studies (cross-sectional studies and cohorts) or clinical trials (randomized and non-randomized); (4) the prevalence of sarcopenia was reported as a percentage or calculated from the results. The exclusion criteria were as follows: (1) the study type was a review, case report, conference abstract, comment, or editorial; (2) sarcopenia is not clearly defined; and (3) the data were obviously incorrect or incomplete.

### 2.3. Data extraction

The full texts of relevant studies were reviewed independently by two authors (JL and YL) after titles and abstract screening. Reference lists of relevant articles were manually searched. A cross-check of the following data was performed independently by two authors (JL and YL): first author, publication year, country, study design, sample size, age, sex, BMI, smoking history, diagnostic criteria for sarcopenia, prevalence of sarcopenia, and clinical impact of sarcopenia: spirometry parameters, dyspnea symptoms, exercise capacity, and mortality risk. Any disagreements were the subject of discussion between the two authors or resolution with the help of a third author (XZ).

### 2.4. Quality assessment

The Newcastle-Ottawa Scale (NOS) was used for cross-section and cohort studies (14). An NOS score of 0–3, 4–6, or 7–9 describes a study as low quality, moderate quality, or high quality, respectively. An independent quality assessment procedure was conducted by two authors (JL and YL), and any disagreements were resolved by a third author (XZ).

### 2.5. Statistical analysis

Effective sizes were extracted and reported where associations were made between sarcopenia and IPF. Articles included in this meta-analysis need to be reported the prevalence of sarcopenia in patients with IPF, or if the article provided sufficient information to calculate the prevalence of sarcopenia in patients with IPF. Data analyses were performed using Stata MP 17.0 (Texas, USA). A random effects model was used to account for differences between articles and the *I*^2^ statistic was used to describe statistical heterogeneities (15). The meta-analysis was graphically represented by forest plots. A meta-regression analyses was used for variables such as sample size, mean age, percentage of males, mean body mass index (BMI), ever smoke, smoking history (packet per year), the percentage value of forced vital capacity (FVC%), the percentage value of first second forced expiratory volume (FEV1%), forced expiratory volume in 1 s/forced volume vital capacity ratio (FEV1/FVC), the percentage value of diffusing capacity for carbon monoxide (DLco%), gender, age, the Gender-Age-Physiology (GAP) score, and the distance of 6-min walk test (6MWD). Moreover, we used meta-regression to explore whether there are differences in comorbidities, such as diabetes, hypertension, malignancy, and cardiac disease. Egger test was used to evaluate publication bias. The trim and fill method was used if publication bias was observed (16).

## 3. Results

### 3.1. Search result

**Figure 1:**
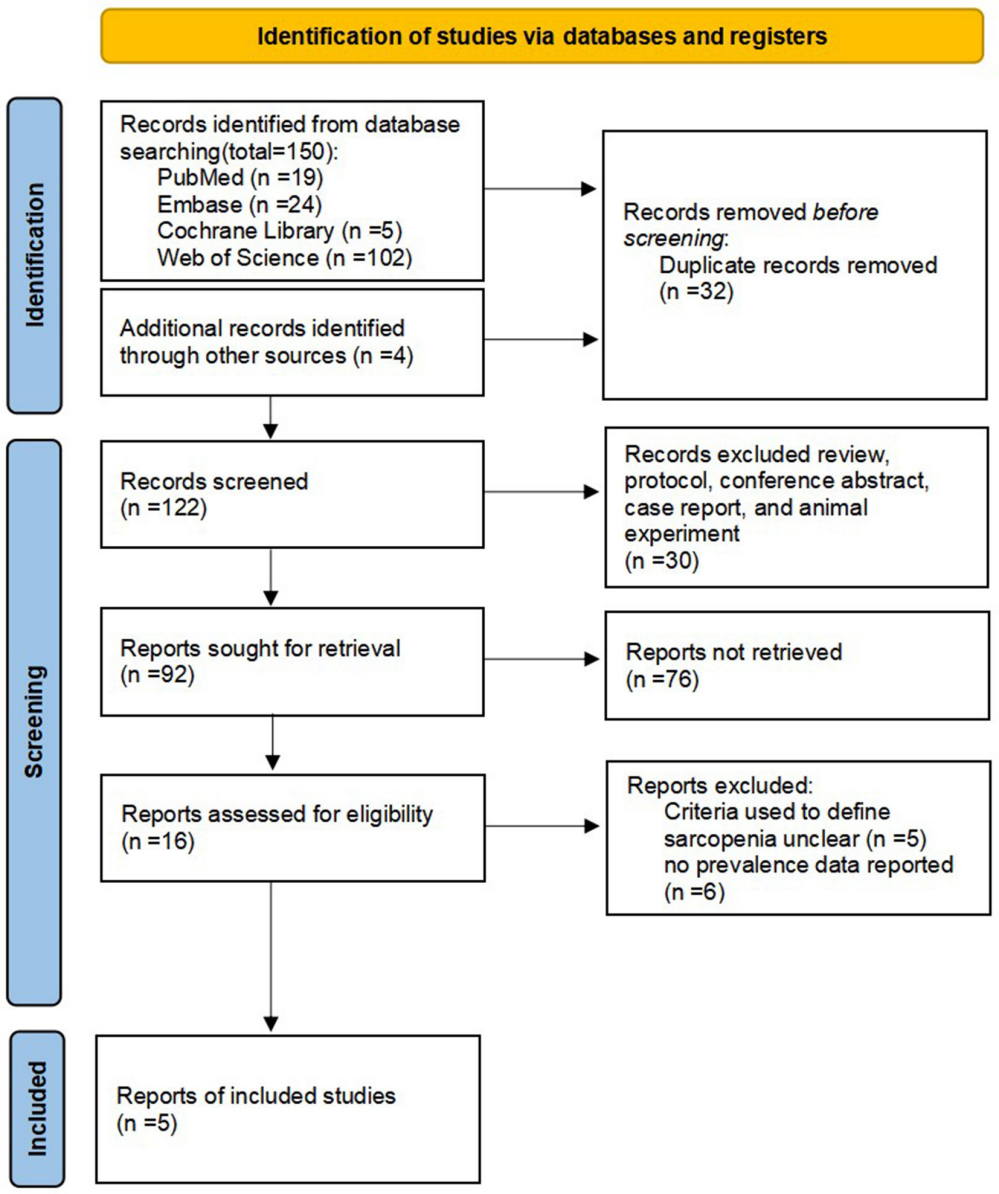
Preferred reporting items for systematic reviews and meta-analyses flow diagram of article selection.

Figure 1 shows a flow chart of the preferred reporting items for systematic reviews and meta-analysis of selected articles. The four databases yielded 154 potential articles. After removing duplicate articles, 122 progressed to title and abstract screening. Of these, 106 articles were excluded, resulting in 16 articles for full-text screening. Ultimately, five publications with 477 participants were included. The overall quality of the included studies was “high” and “moderate” and the prevalence of sarcopenia was reported in all five studies.

Table 1 gives details on the included studies and their sample size, which ranged from 41 to 180. The mean or median age of the study participants ranged from 69.1 to 74.6 years old. There are two cohort and three cross-section publications included in our study. The diagnostic criteria used in the studies were as follows: the lowest quartile of the muscle index at T4 (T4MI), and the lowest quartile of cross-sectional area of the pectoralis muscles (PMcas), the European Working Group on Sarcopenia in Older People 2019 (EWGSOP2 2019) (17), and the Asian Working Group for Sarcopenia 2019 (AWGS 2019) (18). Most of the studies were conducted in Asia (four studies). A summary of the diagnostic criteria used to assess sarcopenia in these included studies is presented in Table 2.

**Table 1:** Characteristic of the included studies regarding the prevalence of sarcopenia in subjects with idiopathic pulmonary fibrosis.

**References/ country**	**Study design**	**Sample size**	**Age, y (mean ±SD)**	**Male *n* (%)**	**BMI, kg/m^2^ (mean ±SD)**	**Diagnostic criteria of sarcopenia**
Moon et al. (9)/Korea	Cross-section	180	69.1	143 (79.4%)	23.9 ± 3.2	(The lowest quartile of T4MI) Q4
Fujikawa et al. (6)/Japan	Retrospective cohort	117	74.6 ± 7.8	30 (25.6%)	22.9 ± 3.6	(The lowest quartile of PMcas) Q4
Faverio et al. (5)/Italy	Prospective cohort	83	72.5 ± 6.9	67 (80.72%)	27.6 ± 4.0	EWGSOP2 2019
Fujita et al. (7)/Japan	Cross-section	56	73.1 ± 7.7	49 (87.5%)	22.3 ± 3.1	AWGS 2019
Hanada et al. (8)/Japan	Prospective cross-section	41	-	-	-	AWGS 2019

**Table 2:** Criteria and cut-off points to diagnose sarcopenia in individuals with IPF in the different studies.

**Criteria**	**Main criteria definition**	**Lean muscle mass**	**Muscle function**					**References**
			**Handgrip strength**	**Gait speed**	**SPPB**	**TUG**	**FTSST**	
EWGSOP2	Low muscle strength + low muscle mass; severity of sarcopenia involve low physical performance	ASMI: Male < 7.0 kg·m^−2^ Female < 5.5 kg·m^−2^	Male < 27 kg Female < 16 kg	≤ 0.8 m·s−1	≤ 8	≥20 s		(5)
AWGS 2019	Low ASM + low muscle strength/low physical performance; severe sarcopenia: low ASM + low muscle strength + low physical performance	ASM: DXA (M: < 7.0 kg·m^−2^, Female < 5.4 kg·m^−2^) BIA (M: < 7.0 kg·m^−2^, Female < 5.7 kg·m^−2^)	Male < 28 kg Female < 18 kg	< 1 m·s^−1^ (6MWT)	≤ 9		≥12 s	(7, 8)
The lowest quartile of T4MI (Q4)	Patients' T4MI ranged from minimum to 25%							(9)
The lowest quartile of PMcas (Q4)	Patients' PMcas ranged from minimum to 25%							(6)

SPPB, Short Physical Performance Battery; TUG, timed up and go test; FTSST, Five Times Sit-to-Stand Test; EWGSOP2, European Working Group on Sarcopenia in Older People 2019; ASMI, appendicular skeletal muscle mass index; AWGS 2019, Asian Working Group on Sarcopenia in 2019; ASM, Appendicular skeletal muscle mass; DXA, Dual-energy X-ray absorptiometry; BIA, Bioelectrical impedance analysis; 6MWT, 6-min walk test; T4MI, the muscle index at T4; PMcas, cross-sectional area of the pectoralis muscles.

### 3.2. Meta-analysis

**Figure 2:**
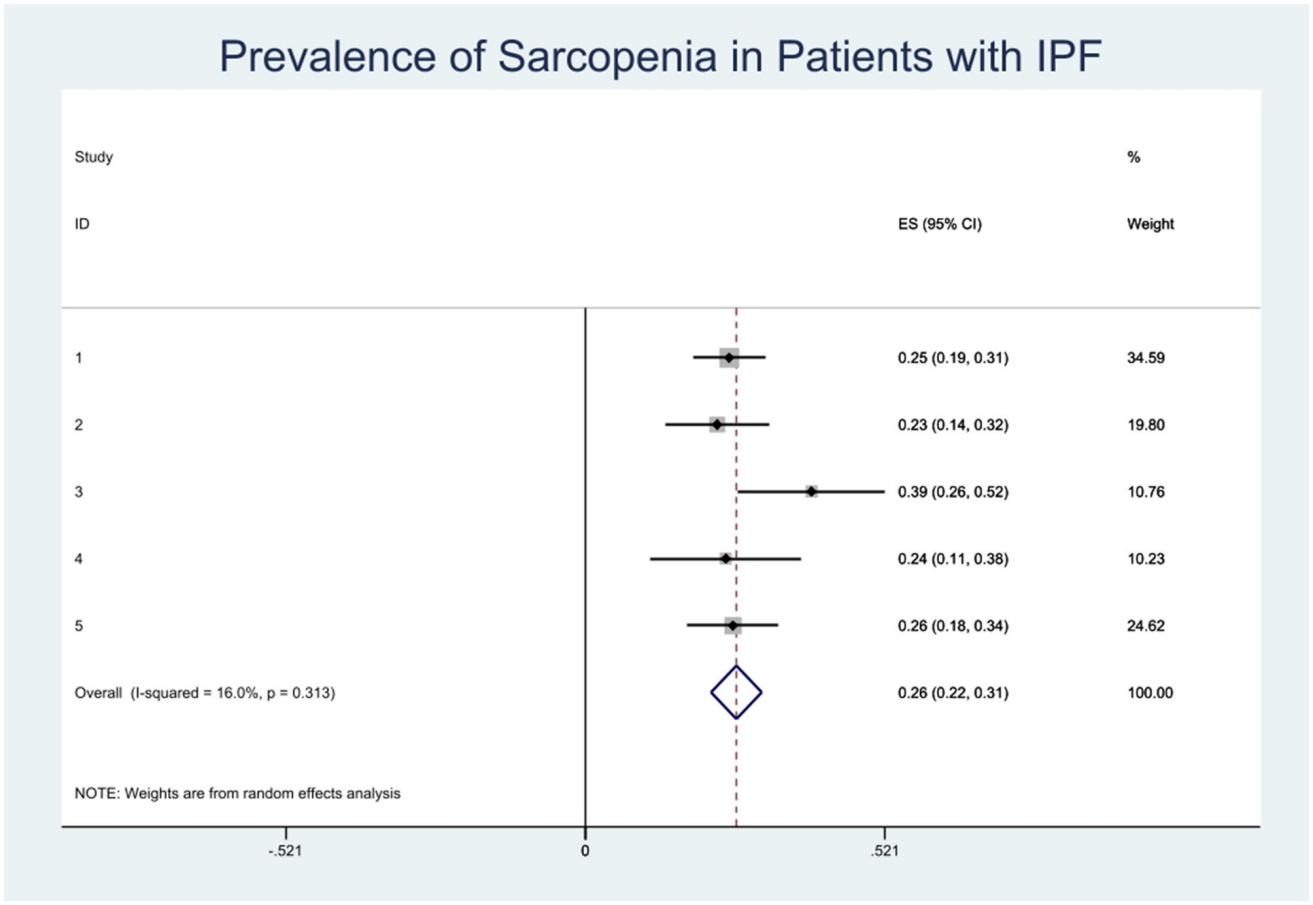
Pooled prevalence and the result of heterogeneity test.

**Figure 3:**
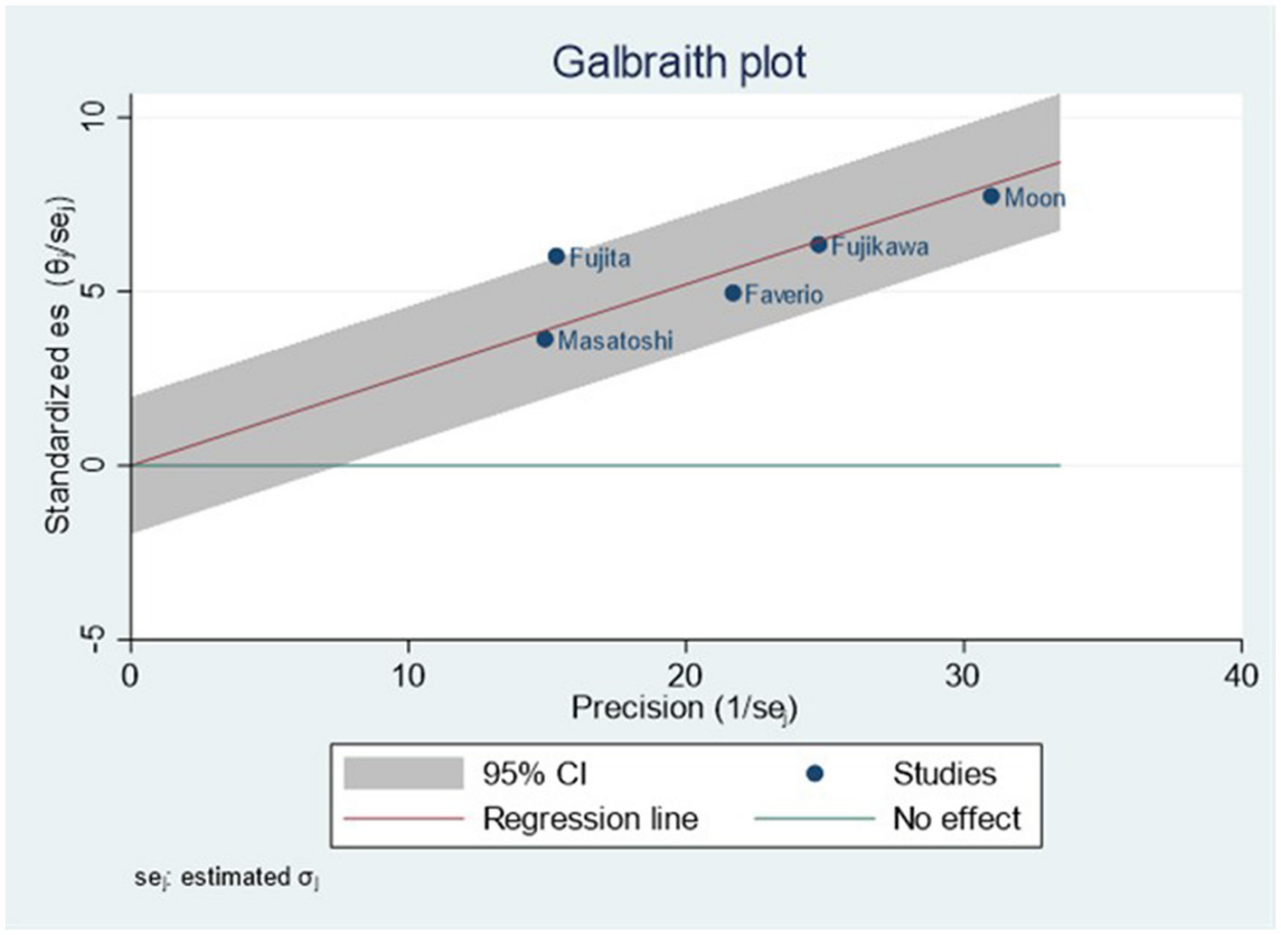
The result of sensitivity analysis.

The combined prevalence of sarcopenia in patients with IPF was 26% (95% CI, 0.22–0.31). No significant heterogeneity was observed among the studies included in this meta-analysis (*I*^2^ = 16.0%) (Figure 2). We carried out sensitivity analysis on included articles and the group mean of four articles fell within the 95% confidence interval of the population, indicating that only one of the five groups of data included had a different impact on the overall results (Figure 3).

In the meta-regression analysis, age, BMI, FVC%, FEV_1_%, DLco%, and GAP score were associated with sarcopenia prevalence among patients with IPF (*p* < 0.05). Demographically, patients with sarcopenia have a higher mean age (*p* = 0.0131) and lower BMI (*p* = 0.001); In terms of pulmonary function test results, patients with sarcopenia had lower FVC% (*p* < 0.001), FEV_1_% (*p* = 0.006), and DLco% (*p* < 0.001). Moreover, patients with sarcopenia were more likely to have a higher GAP score (*p* = 0.003). However, no significant differences were observed between these two groups (IPF patients with sarcopenia, and IPF patients without sarcopenia) with regards to gender (*p* = 0.630), ever smoke (*p* = 0.063), smoking history (pack/year) (*p* = 0.109), FEV_1_/FVC (*p* = 0.069), or 6MWD (*p* = 0.189). After analyzing the available data, we did not find which comorbidities were more common in IPF patients with sarcopenia, no significant difference was found in terms of diabetes (*p* = 0.995), hypertension (*p* = 0.44), malignancy (*p* = 0.554), and cardiovascular disease (*p* = 0.554).

The results of Egger test showed that the publication bias is low (*p* = 0.2660).

## 4. Discussion

This meta-analysis demonstrated that the overall prevalence of sarcopenia in patients with IPF is 26%, and the all-inclusive prevalence of sarcopenia in patients with IPF ranged from 23 to 39%. The highest was observed in a study by Fujita et al. (7) from Japan. The group mean was outside the 95% confidence interval of the population in sensitivity analysis. It is known that the prevalence of sarcopenia increases with age, but in the Fujita et al. study (7) the mean age was older than others, so differences in the included patients' characteristics led to the high prevalence.

We found certain deficiencies in the included studies. First, they used different diagnostic criteria in identifying sarcopenia which could affect the prevalence. A meta-analysis also explored definition can affect the prevalence of sarcopenia (19). Second, we could not acquire the proportion of each age group of these included studies, the prevalence of IPF and sarcopenia all increases with age (20, 21), retired people used to be older and are less physically active. Third, people who were in serious condition and who cannot take pulmonary function or muscle mass tests were not represented in these studies. What's more, prevalence also depends on the setting, and it is more often seen in patients in hospital, post-acute care settings or care homes than in the community (22). These differences can lead to an underestimation in the prevalence of sarcopenia in patients with IPF.

In our study, age, BMI, FVC%, FEV1%, DLco%, and GAP score were considered as risk factors of sarcopenia in patients with IPF. We found that older patients with IPF were more likely to have sarcopenia, consistent with a previous study (23), which also suggested that the prevalence of sarcopenia is as high as 15% in patients over the age of 65 and 50% in people over the age of 80. Thus, when faced with aged patients with IPF, sarcopenia should be considered. In terms of lung function, patients with sarcopenia have worse FVC, FEV1, and DLco. Decreased lung function and symptoms of dyspnea can lead to a reduction in physical activity, which may lead to atrophic muscular disorder (24). In turn, the decrease in muscle mass and function can also lead to poor lung function and dyspnea, creating a vicious cycle. In our study, patients in the sarcopenia group had a higher GAP score, which is the same in studies by Faverio et al. (5) and Fujita et al. (7). As GAP score is the most commonly accepted prognostic scoring system, IPF patients with sarcopenia have a higher GAP score and a poorer prognosis (25).

There were no significant differences in gender, ever smoke, smoking history (pack/year), FEV1/FVC, and 6MWD. A previous study (9) divided and analyzed male and female patients separately as male and female patients exhibited significant differences in muscle mass, but we could not do this as the information was insufficient and some criterion for the diagnoses of sarcopenia were different between males and females. A cohort study (26) has showed that IPF is a male dominant disease, and males may develop IPF earlier because they are more exposed to fibrotic triggers. In our included studies, males also account for the majority of patients, but there is no significant difference in the prevalence of sarcopenia in male and female IPF patients. Further studies are needed to access the statistical data and understand the mechanisms that may account for the sex differences (27). The 6MWD is commonly used as a test to objectively assess the functional exercise capacity of patients with moderate to severe lung disease (28). In patients with IPF, decreased pulmonary diffusion function has a strong impact on exercise tolerance, while the impact of reduced muscle mass and function on exercise tolerance is negligible.

Chronic diseases were recognized as risk factors of sarcopenia. However, we did not found significant difference in terms of diabetes, hypertension, malignancy, and cardiac disease between these two groups (IPF patients with sarcopenia and IPF patients without sarcopenia). This may be due to the different types of diseases targeted by each study, and there is still not enough relevant data.

As for anti-fibrosis drugs (such as nintedanib and pirfenidone) on sarcopenia, statistical analysis could not be made in the enrolled studies, because the definitions of treatment were not clear enough. The use of nintedanib or pirfenidone can lead to anorexia and gastrointestinal disturbance (29), which may effect anti-fibrosis therapy and nutritional health in patients with IPF. A recent study by West et al. declared that adverse events after inhaled pirfenidone were all mild or moderate (30), which indicated that inhaled pirfenidone may lessen the adverse impact of anti-fibrotic therapy.

In sarcopenia, nutrient supplementation have been shown to be effective interventions. Nutritional supplementation have been shown to benefit muscle strength and exercise tolerance in patients with chronic obstructive pulmonary disease (31). An RCT trial has demonstrated that a vitamin D and leucine-enriched whey protein oral nutritional supplement can improve muscle mass and lower-extremity function among sarcopenic older adults (32). Another study showed that astaxanthin improves muscle strength in healthy elderly in addition to the elevation in endurance and walking distance (33). It seems that additional nutritional supplements are also beneficial for IPF patients to prevent sarcopenia. Further studies are needed to explore the types, dosages, and safety of nutrient supplements.

In our study, we found no significant difference in the prevalence of sarcopenia in patients with IPF among the included studies and the publication bias was low which indicate the included studies have good homogeneity. Considering the physical condition of patients who can participant in these clinical trials were fair (mean FVC% 71.34–86%, mean DLco% 46.8–71%, mean 6MWD 352.77 ± 97.65 m), it is easy to under-estimate the prevalence of sarcopenia in patients with IPF for enrollment studies. For this reason, we should pay more attention to these risk factors in patients with IPF to identify sarcopenia as early as possible.

## 5. Limitations

Our study has some limitations. Firstly, the sample size was small and all the information was collected in hospital settings. Secondly, there is no unified approach to diagnose sarcopenia among different races and populations. Thirdly, the current evidence was insufficient to infer prognosis.

Given these limitations, these results should be interpreted with caution. When assessing the risk of sarcopenia in patients with IPF, differences between study settings (i.e., community residents vs. patients in hospitals or nursing care facilities) should be considered. Larger multiethnic, prospective cohort studies should be designed to evaluate sarcopenia prevalence in patients with IPF.

## 6. Conclusion

This study pooled prevalence of sarcopenia in patients with IPF is 26% and the risk factors for sarcopenia in patients with IPF were age, BMI, FVC%, FEV1%, DLco%, and GAP score. Further researches are needed to determine the relationship between sarcopenia and IPF, and to ensure the development of personalized assessment and treatment strategies.

## Data availability statement

The original contributions presented in the study are included in the article/Supplementary material, further inquiries can be directed to the corresponding author.

## Author contributions

GH conceived this manuscript and was responsible for conceptualization and study design. JL, YL, and XZ conducted the database search, study evaluation, and data extraction. JL and YL conducted the statistical analysis. JL, YL, MD, RT, QZ, YB, JM, and ZW participated in the manuscript writing and revising. All authors interpreted the data analyses, read, and approved the final manuscript.
