# Supplementary material for: Prevalence and risk factors of sarcopenia in idiopathic pulmonary fibrosis: a systematic review and meta-analysis
Source: Front Med (Lausanne). 2023 Jun 8;10:1187760. doi: 10.3389/fmed.2023.1187760 (PMC10285151; doi:10.3389/fmed.2023.1187760)
Supplement: Supplementary file 1 [file Table_1.DOCX]

Supplementary Material

Prevalence and Risk Factors of Sarcopenia in Idiopathic Pulmonary Fibrosis: A Systematic Review and Meta-analysis

Jiaye Li^1†^, Ye Lu^2†^, Mingming Deng^1^, Run Tong^1^, Qin Zhang^1^, Yiding Bian^1^, Jinrui Miao^1^, Zilin Wang^1^, Xiaoming Zhou^3^, Gang Hou^1*^

^†^JL and YL equally contributed to the study

*** Correspondence:** Gang Hou: hougangcmu@163.com

# Supplementary 1

Pubmed：

(((((((((((((((((((((((("Idiopathic Pulmonary Fibrosis"[Mesh]) OR (Idiopathic Pulmonary Fibrosis[Title/Abstract])) OR (Idiopathic Pulmonary Fibroses[Title/Abstract])) OR (Pulmonary Fibroses, Idiopathic[Title/Abstract])) OR (Idiopathic Fibrosing Alveolitis, Chronic Form[Title/Abstract])) OR (Fibrosing Alveolitis, Cryptogenic[Title/Abstract])) OR (Fibrocystic Pulmonary Dysplasia[Title/Abstract])) OR (Dysplasia, Fibrocystic Pulmonary[Title/Abstract])) OR (Fibrocystic Pulmonary Dysplasias[Title/Abstract])) OR (Pulmonary Dysplasia, Fibrocystic[Title/Abstract])) OR (Cryptogenic Fibrosing Alveolitis[Title/Abstract])) OR (Cryptogenic Fibrosing Alveolitides[Title/Abstract])) OR (Fibrosing Alveolitides, Cryptogenic[Title/Abstract])) OR (Pulmonary Fibrosis, Idiopathic[Title/Abstract])) OR (Familial Idiopathic Pulmonary Fibrosis[Title/Abstract])) OR (Idiopathic Pulmonary Fibrosis, Familial[Title/Abstract])) OR (Usual Interstitial Pneumonia[Title/Abstract])) OR (Interstitial Pneumonia, Usual[Title/Abstract])) OR (Usual Interstitial Pneumonias[Title/Abstract])) OR (Interstitial Pneumonitis, Usual[Title/Abstract])) OR (Pneumonitides, Usual Interstitial[Title/Abstract])) OR (Pneumonitis, Usual Interstitial[Title/Abstract])) OR (Usual Interstitial Pneumonitis[Title/Abstract])) AND (((((((("Sarcopenia"[Mesh]) OR (Sarcopenia[Title/Abstract])) OR (Sarcopenias[Title/Abstract])) OR (Muscle atrophy[Title/Abstract])) OR (Sarcopenic[Title/Abstract])) OR (Muscle attenuation[Title/Abstract])) OR (Muscle loss[Title/Abstract])) OR (Muscle depletion[Title/Abstract]))

Web of science:

(TS=(Idiopathic Pulmonary Fibrosis) OR AB=(Idiopathic Pulmonary Fibrosis OR Idiopathic Pulmonary Fibroses OR Pulmonary Fibroses, Idiopathic OR Idiopathic Fibrosing Alveolitis, Chronic Form OR Fibrosing Alveolitis, Cryptogenic OR Fibrocystic Pulmonary Dysplasia OR Dysplasia, Fibrocystic Pulmonary OR Fibrocystic Pulmonary Dysplasias OR Pulmonary Dysplasia, Fibrocystic OR Cryptogenic Fibrosing Alveolitis OR Cryptogenic Fibrosing Alveolitides OR Fibrosing Alveolitides, Cryptogenic OR Pulmonary Fibrosis, Idiopathic OR Familial Idiopathic Pulmonary Fibrosis OR Idiopathic Pulmonary Fibrosis, Familial OR Usual Interstitial Pneumonia OR Interstitial Pneumonia, Usual OR Usual Interstitial Pneumonias OR Interstitial Pneumonitis, Usual OR Pneumonitides, Usual Interstitial OR Pneumonitis, Usual Interstitial OR Pneumonitis, Usual Interstitial OR Usual Interstitial Pneumonitis)) AND (TS=(Sarcopenia) OR AB=(Sarcopenia OR Sarcopenias OR Muscle atrophy OR Sarcopenic OR Muscle attenuation OR Muscle loss OR Muscle depletion))

Embase:

Session Results

.......................................................

No. Query Results Results Date

#8. ('idiopathic pulmonary fibrosis':ab,ti OR 24 31 Dec 2022

'idiopathic pulmonary fibroses':ab,ti OR

'pulmonary fibroses, idiopathic':ab,ti OR

'idiopathic fibrosing alveolitis, chronic

form':ab,ti OR 'fibrosing alveolitis,

cryptogenic':ab,ti OR 'fibrocystic pulmonary

dysplasia':ab,ti OR 'dysplasia, fibrocystic

pulmonary':ab,ti OR 'fibrocystic pulmonary

dysplasias':ab,ti OR 'pulmonary dysplasia,

fibrocystic':ab,ti OR 'cryptogenic fibrosing

alveolitis':ab,ti OR 'cryptogenic fibrosing

alveolitides':ab,ti OR 'fibrosing alveolitides,

cryptogenic':ab,ti OR 'pulmonary fibrosis,

idiopathic':ab,ti OR 'familial idiopathic

pulmonary fibrosis':ab,ti OR 'idiopathic

pulmonary fibrosis, familial':ab,ti OR 'usual

interstitial pneumonia':ab,ti OR 'interstitial

pneumonia, usual':ab,ti OR 'usual interstitial

pneumonias':ab,ti OR 'interstitial pneumonitis,

usual':ab,ti OR 'pneumonitides, usual

interstitial':ab,ti OR 'pneumonitis, usual

interstitial':ab,ti OR 'usual interstitial

pneumonitis':ab,ti) AND (sarcopenia:ab,ti OR

sarcopenias:ab,ti OR 'muscle atrophy':ab,ti OR

sarcopenic:ab,ti OR 'muscle attenuation':ab,ti OR

'muscle loss':ab,ti OR 'muscle depletion':ab,ti)

.......................................................

Cochrane Library

Search Name:

Date Run: 31/12/2022 11:07:39

Comment:

ID Search Hits

#1 MeSH descriptor: [Idiopathic Pulmonary Fibrosis] explode all trees 475

#2 (Idiopathic Pulmonary Fibrosis or Idiopathic Pulmonary Fibroses or Pulmonary Fibroses, Idiopathic or Idiopathic Fibrosing Alveolitis, Chronic Form or Fibrosing Alveolitis, Cryptogenic or Fibrocystic Pulmonary Dysplasia or Dysplasia, Fibrocystic Pulmonary or Fibrocystic Pulmonary Dysplasias or Pulmonary Dysplasia, Fibrocystic or Cryptogenic Fibrosing Alveolitis or Cryptogenic Fibrosing Alveolitides or Fibrosing Alveolitides, Cryptogenic or Pulmonary Fibrosis, Idiopathic or Familial Idiopathic Pulmonary Fibrosis or Idiopathic Pulmonary Fibrosis, Familial or Usual Interstitial Pneumonia or Interstitial Pneumonia, Usual or Usual Interstitial Pneumonias or Interstitial Pneumonitis, Usual or Pneumonitides, Usual Interstitial or Pneumonitis, Usual Interstitial or Usual Interstitial Pneumonitides or Usual Interstitial Pneumonitis):ti,ab,kw (Word variations have been searched) 1489

#3 #1 or #2 1499

#4 MeSH descriptor: [Sarcopenia] explode all trees 742

#5 (Sarcopenia or Sarcopenia or Sarcopenias or Muscle atrophy or Sarcopenic or Muscle attenuation or Muscle loss or Muscle depletion):ti,ab,kw (Word variations have been searched) 12266

#6 #4 or #5 12266

#7 #3 and #6 5
